# Venom-loaded cationic-functionalized poly(lactic acid) nanoparticles for serum production against *Tityus serrulatus* scorpion

**DOI:** 10.3762/bjnano.16.115

**Published:** 2025-09-17

**Authors:** Philippe de Castro Mesquita, Karla Samara Rocha Soares, Manoela Torres-Rêgo, Emanuell dos Santos-Silva, Mariana Farias Alves-Silva, Alianda Maira Cornélio, Matheus de Freitas Fernandes-Pedrosa, Arnóbio Antônio da Silva-Júnior

**Affiliations:** 1 Laboratory of Pharmaceutical Technology and Biotechnology, Department of Pharmacy, Federal University of Rio Grande do Norte (UFRN), Natal, RN, Brazil; 2 Department of Morphology, Federal University of Rio Grande do Norte-UFRN, Natal, RN, Brazil

**Keywords:** cationic nanoparticles, immunoadjuvant, polyethylenimine, poly(lactic acid), *Tityus serrulatus*

## Abstract

Reported accidents involving scorpion poisoning by *Tityus serrulatus* are the most frequent in Brazil. The only specific treatment for envenomation is the administration of antivenoms associated with traditional adjuvants. Novel adjuvants are studied to reduce or avoid side effects and potentialize the efficacy of conventional serum. In this study, poly(lactic acid) nanoparticles were functionalized with polyethylenimine for loading peptides and proteins of *T. serrulatus* venom, and their use as a potential immunoadjuvant was evaluated. The protein loading efficiency of about 100% and the polyacrylamide gel electrophoresis assay confirmed the success of venom loading. Dynamic light scattering and zeta potential analysis supported small and narrow-sized cationic functionalized nanoparticles. Atomic force microscopy and scanning electron microscopy images showed nanoparticles with a spherical and smooth shape. The stability of tested formulations was accessed for six weeks, and the sustained release of proteins controlled by diffusion mechanism was also measured. Finally, in vivo immunization in BALB/c mice showed superior efficacy of the *T. serrulatus* venom protein-loaded nanoparticles compared to the traditional aluminum hydroxide immunoadjuvant. Thus, the formulations shown are promising nanocarriers to be used as a biotechnological approach to immunotherapy against scorpion envenomation.

## Introduction

Accidents caused by scorpion envenoming are recognized as an important public health problem in tropical and subtropical regions, due to the high incidence and/or severity of cases, especially for children and elders, and difficulty of management by public health services [1–4]. In Brazil, although 22 species of the scorpion genus *Tityus* have been described in the country, *Tityus serrulatus* is a Brazilian scorpion species with great medical significance [2,5], responsible for the highest number of accidents and also the most severe ones [6–7].

Scorpion toxins represent a vast collection (≈100,000) of pharmacologically relevant peptide toxins that have provided an important foundation for advancing the studies in this field [8]. *Tityus serrulatus* venom is comprised of several compounds such as mucus, salts, proteins with high molecular mass, nucleotides, lipids, amino acids, hyaluronidase, hypotension factors, metalloproteases, and neurotoxins [7,9]. However, neurotoxins are considered the main responsible for the envenoming syndrome as well as the most studied [10].

With regard to treatment, in severe scorpion envenoming cases, immunotherapy is the most common approach to protect populations from lethal effects [1,10–11]. Aluminum-based adjuvants have been extensively used to induce long-lasting protective immunity through vaccination [12–13]. However, reported incidences of toxicity and side effects of aluminum have raised concerns regarding their safety in childhood vaccines [13]. Consequently, there is a growing need for alternative immunization strategies that not only improve safety but also effectively deliver complex venom proteins.

The bioactive proteins that compound the *Tityus serrulatus* venom are complex molecules that should have the structural integrity preserved for the specific biological activity. The poor stability of these proteins, both in vivo and in vitro, creates a challenge for drug delivery systems aiming to effectively target affected tissues or cells [14–15]. Nanocarriers have been widely studied for enabling prolonged circulation and sustained drug release over time, depending on their structural properties [16–17]. Therefore, protein delivery through nanoparticles is an effective way to control drug release as well as to design an efficient protein delivery system [16].

Among different materials used for nanocarriers, several polymers have been investigated for producing cationic nanocarriers due to their ability to cross biological barriers, their biocompatibility, and low toxicity [18]. Their manipulation at the nanoscale changes specific surface properties, possibly improving the ability to cross biological barriers targeting the affected tissues [18–19].

In this context, nanoparticle controlled release based on biodegradable polymers such as poly(lactic acid) (PLA) has been investigated [13]. The nanoparticles produced using these synthetic polyesters show neutral or negative zeta potential, which limits the loading of negatively charged macromolecules such as proteins, polypeptides, or DNA [14,20]. The surface of nanoparticles can be modified to achieve high protein loading or avoid a rapid cellular uptake. Using different strategies, nanoparticles have been functionalized with a variety of ligands such as small molecules, surfactants, polymers, and biomolecules [21–22].

The use of cationic molecules, as polyethylenimine (PEI), to change the surface of nanoparticles to a positive potential, improving the interaction with negatively charged biomolecules, is one strategy successfully employed for gene delivery [20,23–24]. These cationic nanoparticles have an absent or weak electrostatic interaction with negatively charged peptides, proteins, antigens, oligonucleotides, polypeptides, or DNA [18].

The PLA is well established to produce nanoparticles as carriers for drugs or biomolecules from a biotechnology source due to its natural metabolism pathway [25–26]. In a recent study, PLA was successfully employed to detoxify, preserve antigenicity, and enhance immune protection against scorpion toxins [11]. At the same time, it has always been a constant effort and focus to either search for alternative adjuvants or to reduce the quantity of aluminum in the vaccines. In this direction, controlled release of micro- and nanoparticulate formulations based on biodegradable polymers such as PLA have been investigated [13,27–32].

A more detailed approach for use of a delivery system as a new nontoxic and non-inflammatory immunoadjuvant is of great importance to public health. The present study was designed with the aim to evaluate the effectiveness of biodegradable PLA polymeric nanoparticles functionalized with PEI as an adjuvant and potential candidate for vaccine delivery against *T. serrulatus* venom.

## Results

### Protein loading efficiency of the *Tityus serrulatus* scorpion venom

The *T. serrulatus* venom protein-loaded PLA nanoparticles were fabricated by nanoprecipitation methods. In this technique, the PLA nanoparticles (NPs) were produced by low-energy solvent displacement and functionalized with polyethylenimine (cationic polymer) for the *T. serrulatus* protein adsorption.

The NPs showed a mean diameter of 165 nm and a positive zeta potential of 7.0 mV. After the addition of venom, the PLA nanoparticles loaded with *T. serrulatus* venom proteins remained with a narrow particle size distribution. Moreover, an increase in size of the particles occurred after the addition of the venom for both concentrations (*p < 0.05), without significantly altering the zeta potential (Table 1). A polydispersity index (PDI) smaller than 0.3 was required for all the analyses. The quantification of proteins by the bicinchoninic acid (BCA) assay showed an encapsulation efficiency (EE) of 100% for all samples containing *T. serrulatus* venom (Table 1).

**Table 1:** Encapsulation efficiency (EE), mean diameter and zeta potential of PLA nanoparticles (NPs), *T. serrulatus* venom protein-loaded PLA nanoparticles at 0.5% (NPs + Tsv 0.5%) and *T. serrulatus* venom protein-loaded PLA nanoparticles at 1.0% (NPs + Tsv 1.0%).

Samples	EE (%)	Mean diameter	Zeta potential (mV)

NPs	—	165.0 ± 25.3	7.00 ± 4.10
NPs + Tsv 0.5%	100.0	226.4 ± 13.6*	3.58 ± 1.56
NPs + Tsv 1.0%	100.0	230.9 ± 15.59*	4.40 ± 1.99

Values are the mean ± standard deviation, n = 3; *p < 0.05 for the venom group compared to the NPs group.

**Figure 1:**
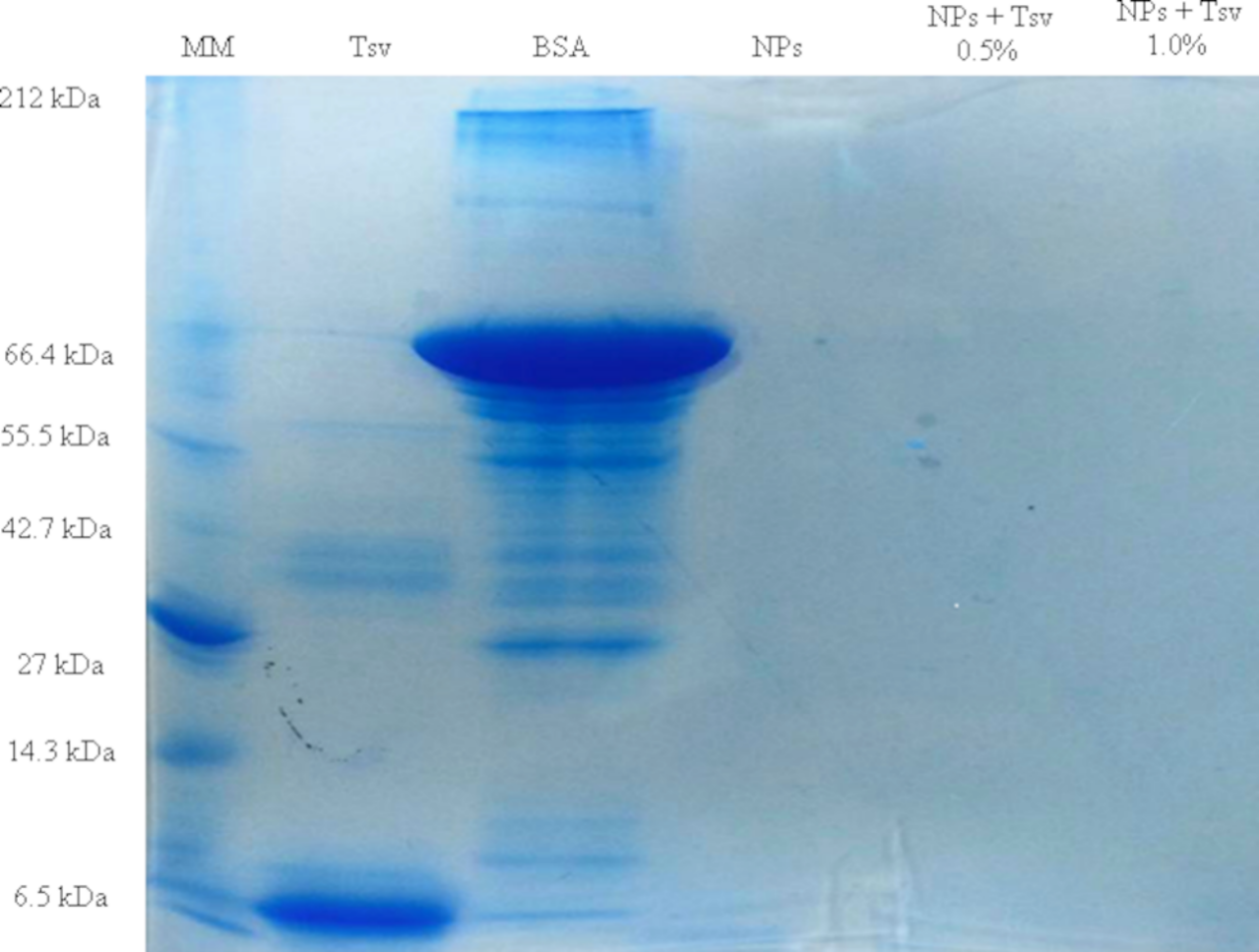
Electrophoretic profile of *Tityus serrulatus* venom (Tsv), bovine serum albumin (BSA), PLA nanoparticles (NPs), *T. serrulatus* venom protein-loaded PLA nanoparticles at 0.5% (NPs + Tsv 0.5%), and *T. serrulatus* venom protein-loaded PLA nanoparticles at 1.0% (NPs + Tsv 1.0%). MM: molecular mass marker. Gel stained with Coomassie brilliant blue R-250.

The high percentage of protein incorporation was confirmed by the electrophoretic profiles in sodium dodecyl sulfate polyacrylamide gel (SDS-PAGE), after the gel was stained with Coomassie brilliant blue R-250. In this methodology, the nanoparticles are retained in the wells and only the proteins that were not incorporated in the nanoparticles will migrate through the gel. The *T. serrulatus* venom (Tsv) protein presented a molecular mass range of approximately 2 to 66.4 kDa. Comparing the electrophoretic profiles of *T. serrulatus* venom protein-loaded PLA nanoparticles at 0.5% and 1.0% (w/w) with *T. serrulatus* venom was possible to evidence that all proteins of the venom were incorporated within the polymeric matrix, since the protein bands were not evidenced in the gel. The PLA nanoparticles and bovine serum albumin (BSA) were used as controls (Figure 1). This assay corroborates the high encapsulation efficiency obtained by the BCA assay.

### Field emission gun scanning electron microscopy and atomic force microscopy analyses

**Figure 2:**
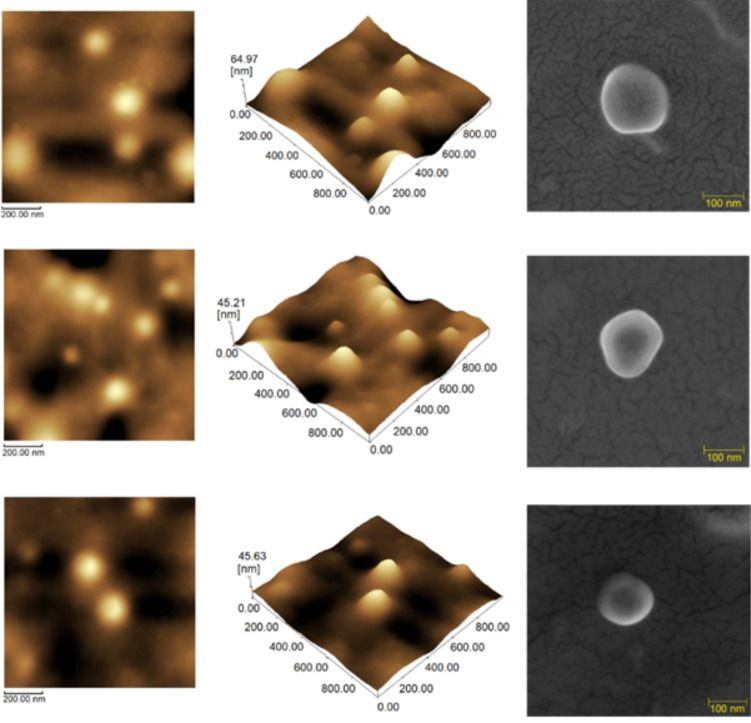
2D and 3D atomic force microscopy and field emission gun scanning electron microscopy images, respectively, of formulations of *Tityus serrulatus* venom-loaded PLA nanoparticles. (A) PLA nanoparticles, (B) *T. serrulatus* venom protein-loaded PLA nanoparticles at 0.5%, and (C) *T. serrulatus* venom protein-loaded PLA nanoparticles at 1.0%.

Field emission gun scanning electron microscopy (FEGSEM) and atomic force microscopy (AFM) analyses were realized to access shape and surface features of NPs and *T. serrulatus* venom protein-loaded PLA nanoparticles at 0.5% and 1.0%. For both techniques, the particles showed uniform characteristics with smooth surface, spherical shape, and great encapsulation efficiency. The addition of *T. serrulatus* venom did not alter the spherical shape, as well as the mean diameters of nanoparticles (Figure 2).

### Physical stability assay

**Figure 3:**
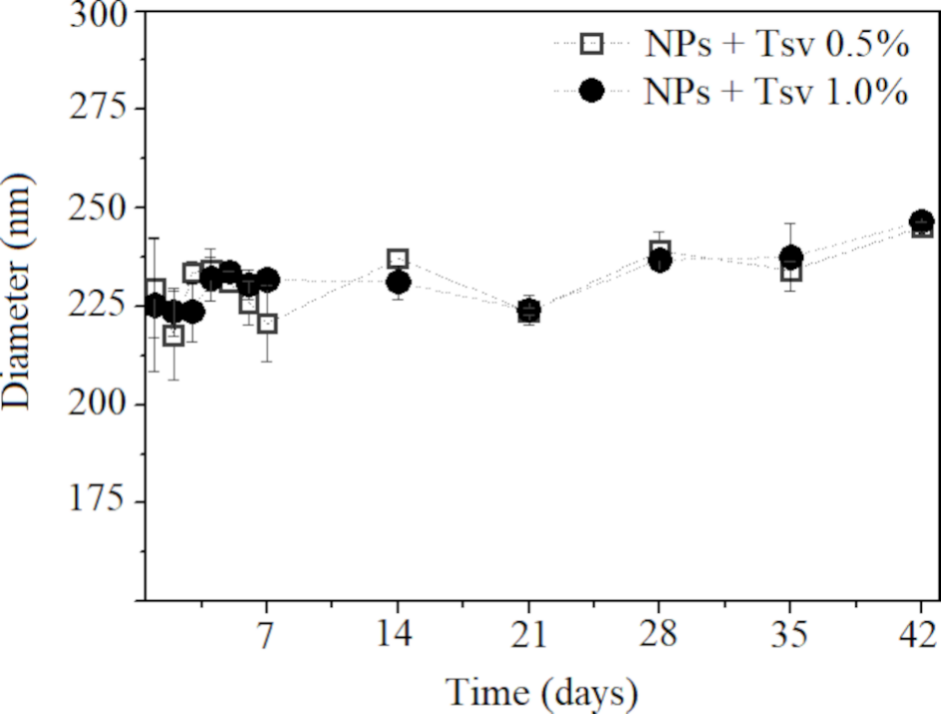
Colloidal stability of *T. serrulatus* venom protein-loaded PLA nanoparticles at 0.5% (NPs + Tsv 0.5%) and *T. serrulatus* venom protein-loaded PLA nanoparticles at 1.0% (NPs + Tsv 1.0%) in the period of six weeks. The data are expressed as mean ± standard deviation.

Distinct NPs with venom-loaded formulations were analyzed for particle size and PDI (Figure 3). The tracking was accomplished for 42 days (six weeks) and the formulations do not show significant differences in particle size (≈225 nm) and PDI (<0.3).

### In vitro protein release

**Figure 4:**
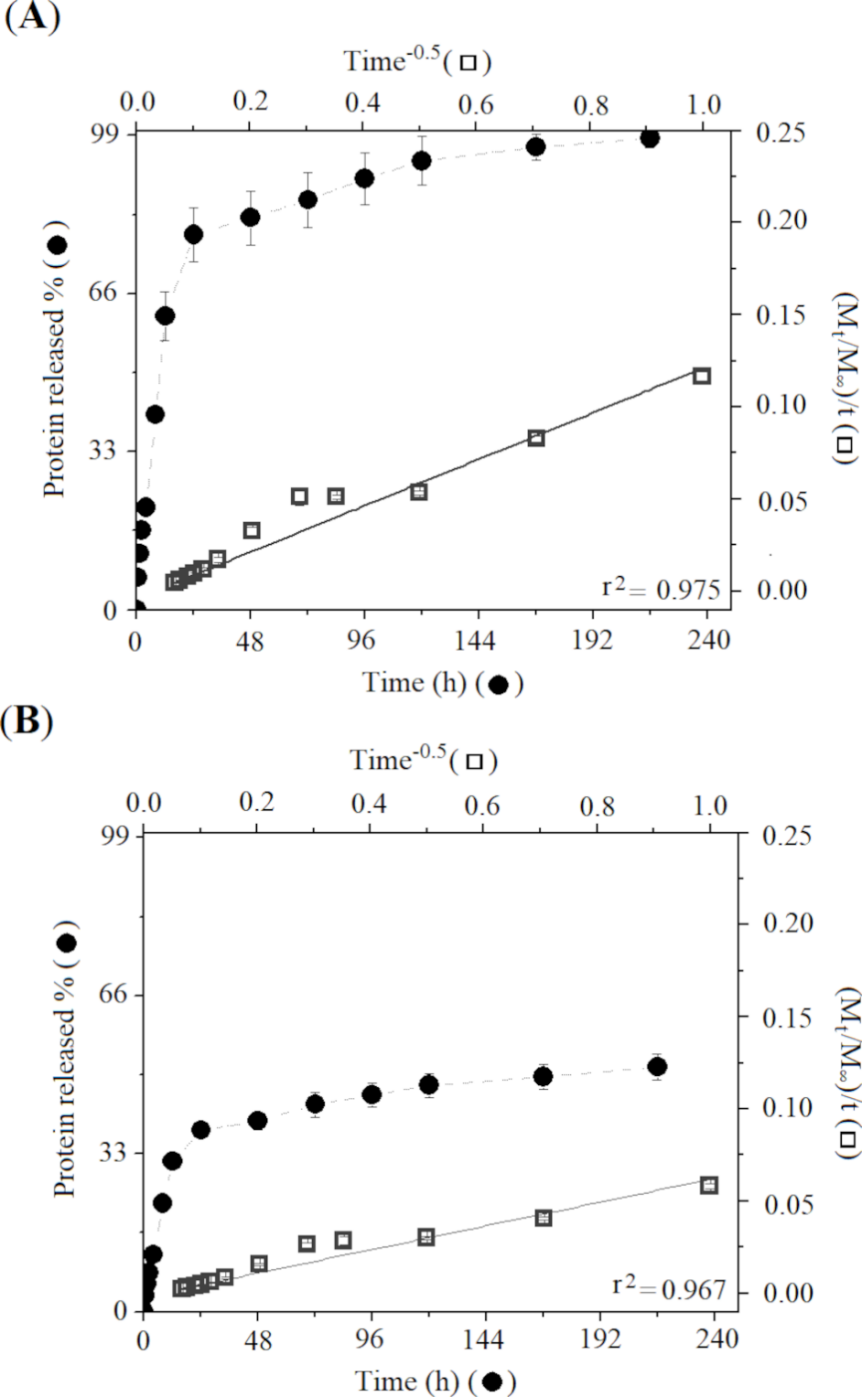
Experimental in vitro release profile of *T. serrulatus* venom-loaded PLA nanoparticles at 0.5, r^2^: 0.975 (A) and 1.0%, r^2^: 0.967 (B). The release profile of each sample is represented by dots. The data are expressed as mean ± standard deviation.

The Figure 4 shows the release profile of *T. serrulatus* venom protein-loaded PLA cationic nanoparticles with two different formulations containing 0.5% (Figure 4a) and 1.0% (Figure 4b) (w/w) of Tsv in the nanoparticle suspension. The in vitro protein release studies showed that all samples exhibited a slight initial burst effect, releasing 30–60% of the total protein mass, followed by the subsequent slow release phase. After 144 h, the *Tityus serrulatus* venom-loaded PLA nanoparticles have released about 88% and 50% of the initial loaded protein for the samples containing 0.5% and 1.0% of *T. serrulatus* venom, respectively.

The mechanism of release of venom protein-loaded PLA nanoparticles profile was studied by applying different mathematical models to the experimental data. The data were subject to four different diffusion kinetic linear models: zero order, first order, Korsmeyer–Peppas, and parabolic diffusion (Table 2). The zero order, first order, and Korsmeyer–Peppas models exhibiting determination coefficient (r^2^) values demonstrate that the release mechanism cannot be explained by these models. The parabolic diffusion model better fitted the venom release from PLA cationic nanoparticles, providing a linear correlation coefficient of 0.975 and 0.967 for 0.5% and 1.0% venom concentrations, respectively.

**Table 2:** Different kinetic mathematical models for the in vitro release *T. serrulatus* venom protein-loaded PLA nanoparticles at 0.5% (NPs + Tsv 0.5%) and *T. serrulatus* venom protein-loaded PLA nanoparticles at 1.0% (NPs + Tsv 1.0%).

Kinetic models	NPs + Tsv 0.5% (r²)	NPs + Tsv 1.0% (r²)

zero order	0.701	0.648
first order	0.412	0.401
Korsmeyer–Peppas	0.940	0.922
parabolic diffusion	0.975	0.967

### Serum antibody responses

**Figure 5:**
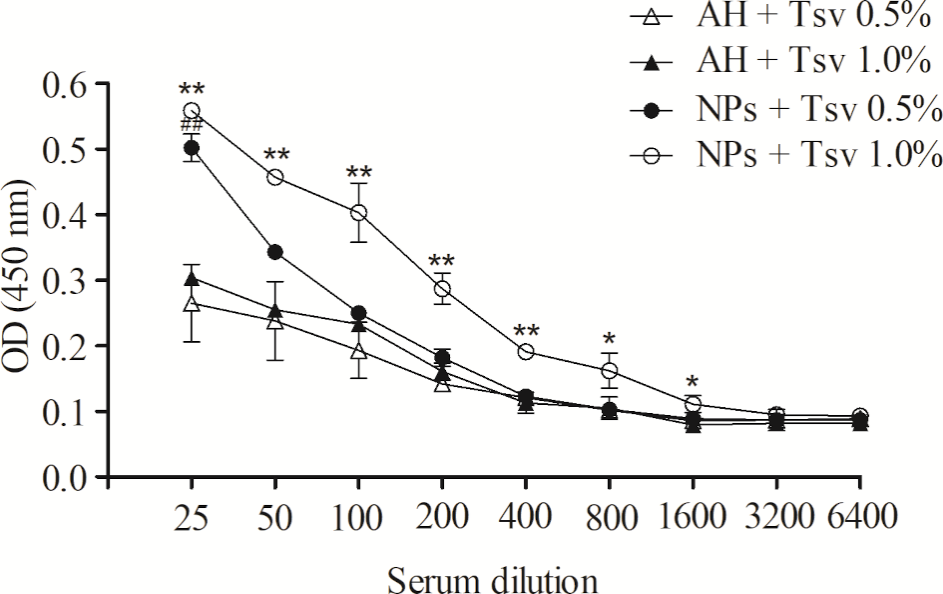
Evaluation of antibody production from mice immunized subcutaneously for six weeks with *Tityus serrulatus* venom-loaded NPs or AH at concentrations of 0.5% or 1.0% determined by ELISA. The data are expressed as mean ± standard deviation. **p < 0.01 and *p < 0.05 compared to NPs + Tsv 1.0% group with AH + Tsv 1.0%. ^##^*p* < 0.01 compared to NPs + Tsv 0.5% group with AH + Tsv 0.5%.

The immunization protocol was based in subcutaneous administration for six weeks with 100 µL of venom-loaded NPs or aluminum hydroxide (AH) at concentrations of 0.5% or 1.0% (w/w). The blood samples were treated and subjected to serial dilutions with a PBS/BSA 0.1% solution, and the antibody production was evaluated by enzyme-linked immunosorbent assay (ELISA). The antibody dilutions were detected in the serum of mice immunized with NPs and AH, both venom loaded. At first, NP formulations can produce more antibodies than AH formulations when analyzed in the first dilution (1:25), **p < 0.01. Venom-loaded nanoparticles at 1.0% showed higher effectiveness when compared to venom-loaded AH at 1.0%, while the nanosystems at 0.5% demonstrated to be equipotent to AH 0.5%. The NP formulation produced antibodies that were detected until the 1:3,200 dilution, whereas the AH formulation produced antibodies that were detected until the 1:1,600 dilution. The Figure 5 shows the antibody production results from mice immunized with venom-loaded nanoparticles and aluminum hydroxide, demonstrating higher effectiveness of NPs at a 1.0% concentration compared to that of AH. Nanoparticle results were statistically different to those of the AH immunized groups, and demonstrated that the nanoparticles can stimulate the immune system with low concentration of antigens/venom.

## Discussion

Figure 1 shows the electrophoretic profiles in an SDS-PAGE gel, which suggests high percentage of protein incorporation in cationic nanoparticles and total encapsulation. It is possible to observe the absence of any free protein migrate through the gel for *T. serrulatus* venom protein-loaded PLA nanoparticles at 0.5% and 1.0% (w/w) compared with non-encapsulated proteins. This achievement corroborates with encapsulation efficiency experiments. In the present approach, PLA nanoparticles loaded with *Tityus serrulatus* venom proteins were successfully obtained using parameters selected for the nanoprecipitation method. This low-energy technique allowed the spontaneous self-assembling of PLA nanoparticles, which were functionalized with polyethyleneimine (PEI) to enable the adsorption of venom proteins. Experimental results demonstrated the potential for use in therapeutic serum production against *T. serrulatus*, one of the most dangerous scorpions in South America.

Poly(lactic acid) nanoparticles show neutral or negative zeta potential, which limits the loading of negatively charged species [33]. For this, the surface of the PLA nanoparticles was functionalized with PEI, a hydrophilic polymer that provides a positive surface charge. This modification enhances high macromolecule loading or improves the cell interaction and cellular uptake [22,33–34]. The cationic character induces biomolecules with negative charges, such as proteins, peptides, DNA, RNA, antigens, and oligonucleotides to be efficiently incorporated through electrostatic interactions [35–36]. Moreover, some studies showed the interesting association of nanoparticles containing PEI for incorporation of DNA in gene transfection and BSA protein [14,37].

The physicochemical characterization of the nanoparticles revealed mean diameters below 230 nm, positive zeta potentials, and PDI below 0.3, corroborating other reports of PLA–PEI systems [14,38]. In addition, the high encapsulation efficiency can be attributed to the functionalizing agent (PEI), which demonstrates a certain advantage over the polymer, and it is responsible for the nanoparticle cationic character, ensuring binding of venom proteins. Similar results were obtained using the ionic affinity interaction to bind negatively charged proteins of the *T. serrulatus* scorpion venom to the amine groups of cationic nanoparticles prepared by ionic gelation with 100% encapsulation efficiency [1–2]. Additionally, PEI with high branching density has been shown to enhance complexation and transfection efficiency [23].

The morphology obtained by AFM and FEGSEM showed particles with a smooth surface, spherical shape, uniform aspects, and sizes compatible with the results obtained by dynamic light scattering. These morphological features are known to influence the biological activity of nanoparticles loaded with bioactive molecules, directly affecting their release. The release profile of a substance depends on the particle size; thus, small particles have a larger surface area for dissolution, providing faster release kinetics [39]. Uniformity of particle size is also important for the stability of the formulation, as well as for the choice of administration route. The intravenous and intramuscular routes are very common for the administration of proteins with pharmacological activity and with particle sizes of around 200 nm [40].

Regarding stability, only minimal changes in physicochemical parameters were observed, indicating colloidal stability of the nanoparticle formulation. This is particularly relevant as nanoparticles have a natural tendency to agglomerate, often leading to the formation of larger aggregates [41]. It is interesting to report that the presence of electrostatic charges on the surface of particles plays a crucial role in maintaining physical stability. However, long-term colloidal stability observed in the formulations with low zeta potential values can be attributed to steric stabilization provided by poly(vinyl alcohol) (PVA) and an additional electrostatic repulsion introduced by surface-bound PEI. This behavior is consistent with previous reports on nanoprecipitation systems that utilize surfactants [26]. The stability of venom-loaded nanoparticles did not exhibit significant changes in particle size, which can be explained by the complex interplay of charges derived from the polycation (PEI) and venom proteins. The net repulsive forces between particles offer a barrier to aggregation and support long-term colloidal stability [14,37].

The protein release study of venom-loaded PLA cationic nanoparticles occurred in two stages. In the first stage, a burst release effect was due to surface-adsorbed proteins, followed by the diffusion through the external swollen layer of particles, and a slow release equilibrium state was demonstrated for distinct formulations [1]. Successful protein-loaded PLA cationic nanoparticles, previously demonstrated, ensured the desired slow-release protein profile effect for all tested formulations. As shown in Figure 4A and Figure 4B, based on the analysis of the determination coefficient (r²), obtained from the application of four mathematical models to experimental data, the model that explains the release profile of the venom-loaded PLA nanoparticles has been identified, and a more accurate adjustment was observed for the parabolic diffusion model. This mathematical model suggests that the release mechanism of venom-loaded PLA nanoparticles was controlled by diffusion dependent on the protein-loading level [18]. These results corroborate with previous studies that used nanoparticles for venom protein delivery [42–43].

During an immune response against poisoning, some toxins are poorly immunogenic, and due to that, they are associated with immunoadjuvants. Information about the efficiency of immunoadjuvants can be accessed by immunization protocols [44]. Venom-loaded NPs obtained better efficiency in the stimulation to the immune system when compared to that of AH. Similar results were found in experiments performed by Ayari-Riabi (2016) using a PLA nanoparticle formulation to stimulate immune response against the venom from *Androctonus australis hector* and *Buthus occitanus tunetanus* scorpions. However, PLA nanoparticles had the same response when compared with that of AH standard formulation [11]. Cationic PLA nanoparticles produced in this study demonstrated better immune stimulation behavior. These results can be found in other chitosan-based cationic nanoparticle formulations [1,45]. The cationic properties probably explain the immune response improvement since different nanoparticle types generated stronger Th1 and Th2 immune responses compared to other antigen types [45].

Although the present study provides physicochemical and immunological data supporting the potential of PLA–PEI nanoparticles as antigen carriers, further investigation is required to assess their safety profile. In vitro cytotoxicity assays on relevant cell lines, particularly immune or epithelial cells and long-term biocompatibility studies, including histopathological analysis following repeated administration, will be essential to evaluate the potential of these nanosystems. These aspects are part of our ongoing research and will be addressed in future studies, aiming to support the translational advancement and industrial applicability of this nanoplatform.

## Conclusion

In conclusion, the enhanced immunoadjuvant effect by functionalized cationic PLA nanoparticles adsorbed with negatively charged proteins was successfully developed by low-energy solvent diffusion method, producing effective and stable spherical cationic nanoparticles. The positive surface charge enabled a high protein incorporation into particles. The prolonged release effect showed a slow release of venom-loaded nanoparticles. Moreover, the biological efficacy of the nanosystems showed that cationic nanoparticles can stimulate the immune system to increase the immune response against *T. serrulatus* venom when compared to the most used immunoadjuvant, aluminum hydroxide. Thus, all data demonstrate a good performance of the nanoprecipitation method to generate small-sized protein-loaded polymeric nanoparticles which can be used as a novel immunoadjuvant.

## Experimental

### Material

Poly(D,L-lactic acid) (D,L-PLA) 50:50 (inherent viscosity 0.63 dL·g^−1^ at 30 °C) was purchased from Birmingham Polymers Inc. (Birmingham, United States of America). Poly(vinyl alcohol) with molecular weight of 30 to 70 kDa and 86.5 to 89.5 kDa when hydrolyzed, hyper-branched PEI with average molecular weight of 25 kDa, BSA, and aluminum hydroxide were purchased from Sigma-Aldrich^®^ (Saint Louis, Missouri, United States of America). The BCA Protein Assay Kit was purchased from Pierce Biotechnology (Woburn, Massachusetts, United States of America) and mouse IgG total ELISA was purchased from eBioscience (San Diego, California, United States of America). Purified water (1.3 µS·cm^−1^) was prepared from a reverse osmosis purification equipment (model OS50 LX, Gehaka, São Paulo, Brazil). All other reagents were of analytical grade.

### Venom

Lyophilized *Tityus serrulatus* scorpion venom was generously supplied by the Instituto Butantan, São Paulo, Brazil. The scientific use of the biological material was approved by the Brazilian Access Authorization and Dispatch Component of Genetic Patrimony (CGen) (Process 010844/2013-9, 25 October 2013). The venom was weighed and dissolved with PBS at 1 mg/mL, aliquoted, and stored at −20 °C until used.

### Preparation of cationic PLA nanoparticles for *Tityus serrulatus* venom delivery

In a manner similar to [14], PLA NPs) were prepared by nanoprecipitation, up to a 30:70 (%) ratio of organic and aqueous phase (OP:AP). The organic phase was set up with 6 mL of acetone solution containing PLA (0.1% w/v) and PEI (0.1% w/v) to 14 mL of the aqueous phase containing PVA (1.0% w/v), at an output flux of 3.0 mL⋅min^−1^. After titrations, the acetone organic solvent evaporation occurred under a 700 rpm of magnetic stirring overnight. After solvent evaporation, colloidal dispersions were centrifuged at 22,000*g* for 5 minutes. The *T. serrulatus* venom loading in the cationic nanoparticles was tested at two different formulations containing 0.5% and 1.0% (w/w) of Tsv in the nanoparticle suspension. The venom aqueous solution was added to the colloidal dispersion containing the cationic-functionalized NPs at 22 °C, which remained under magnetic stirring at 360 rpm for 5 h.

### Physicochemical aspects of nanoparticles

The mean particle size, PDI, zeta potential, and stability of the nanoparticles were assessed by using the cumulative analysis method, according to the intensity of DLS with a particle size analyzer (ZetaPlus, Brookhaven Instruments Corporation, New York, United States of America), equipped with a 90 Plus/BI-MAS apparatus, at a wavelength of 659 nm with a scattering angle of 90°. For the analysis, 100 μL of the nanoparticle suspension was diluted in 900 μL of deionized water (1:10 dilution) to ensure dispersions within a suitable experimental range (100–500 kcps). For the colloidal stability assay, the nanoparticles were stored at a temperature of 4.0 ± 2.0 °C, and every seven days particle sizes were analyzed during six weeks. A polydispersity index smaller than 0.5 was required for all the experiments. All analyses were performed in triplicate and data expressed as mean ± standard deviation.

The shape and surface of nanoparticles were assessed by AFM (SPM-9700 Shimadzu, Tokyo, Japan) and FEGSEM (Zeiss Microscopy, Auriga, Jena, Germany) images. For the AFM analyzes, a drop of dispersion was placed on a clean microscope slide and dried under a desiccator for 24 h. The images were obtained with a silicon tip, operating in the attractive region of a cantilever in non-contact mode. For the FEGSEM analyzes, a drop of dispersion was placed in a microscope slide with carbon tapes and dried under a desiccator for 24 h [46].

### Protein-loading efficiency

The different concentration samples of protein-loaded PLA nanoparticles were carefully transferred to 1.5 mL centrifuge tubes, and then centrifuged at 20,000*g* at 4 °C for 30 min. The protein concentration of the supernatant was quantified using a BCA Protein Assay Kit (Thermo Fisher Scientific) according to the manufacturer recommendations, and a microplate reader (Epoch, BioTek^®^, Winooski, Vermont, United States of America) at 562 nm [47] was used. The encapsulation efficiency performed was calculated using Equation 1. All analyses were performed in triplicates and data expressed in percentage.


[1] $$\text{EE}\%\;=\;\left[\frac{\text{total proteins}–\text{free proteins}}{\text{total proteins}}\right]\;\times \;100,$$

where “total proteins” is the total protein amount added and “free proteins” is the non-entrapped protein in the supernatant after centrifugation.

### Sodium dodecyl sulfate in polyacrylamide gel electrophoresis

The electrophoretic profile of *T. serrulatus* venom, free-protein nanoparticles, and different concentrations of protein-loaded nanoparticles were recorded by SDS-PAGE electrophoresis using a minigel system (Mini-Protean^®^ II, BIO-RAD, Hercules, California, United States of America). The molecular weight markers were commercially obtained (Gibco-BRL Life Technologies, Gaithersburg, Maryland, United States of America). The gel was stained with Coomassie Brilliant Blue R-250 solution and scanned [48].

### In vitro protein release

To monitor the protein release profile, 1.5 mL of venom-loaded nanoparticles (0.1% PLA w/v, 1% venom w/w) were suspended in 1 mL of phosphate buffer solution (pH 7.4, KH_2_PO_4_ 0.05 mol⋅L^−1^), in a thermostatic bath (SL-150/22; Solab, Piracicaba, Brazil) at 37 °C. At predetermined time intervals, 100 μL of the suspension was collected and centrifuged at 16,000*g* for 30 min to sediment any residual particles. The supernatant was carefully removed for analysis, and the total protein released was quantified using the BCA Protein Assay Kit (Thermo Fisher Scientific), following the manufacturer instructions. After each collection, the same volume of fresh buffer was added to the tube to maintain constant volume and sustain sink conditions throughout the experiment [14].

### Animals

BALB/c mice (about 30 g, 6–8 weeks old), from both sexes were used for the studies. The animals were maintained at 22 ± 2 °C and in a 12 h dark/12 h light cycle, with free access to standard laboratory chow and water. Each experimental group was composed of five animals (*n* = 5). After the experiments, all animals were euthanized with an overdose of thiopental (100 mg/kg) associated with an intraperitoneal injection of lidocaine 2% (10 mg/kg) followed by cervical dislocation. The experimental protocol was approved by the Committee for Ethics in Animal Experimentation of the Federal University of Rio Grande do Norte (UFRN), Brazil (Protocol No. 015/2015).

### Immunization

Mice received 100 µL of PBS, free-cationic nanoparticles, aluminum hydroxide, *Tityus serrulatus* venom (0.5 or 1.0% w/w) loaded nanoparticles or associated with AH. The immunization was performed once per week for six times via subcutaneous administration in the lumbar region [2].

### Serum production

Blood samples in the absence of anticoagulants were incubated at 37 ºC. After 30 min, the samples were incubated at 4 ºC for 2 h and then were centrifuged at 15,000*g* at 4 ºC for 5 min. The supernatant (serum) was then collected and stored at −20 ºC.

### Antibody titer evaluation

The antibody titers were determined according to Fernandes-Pedrosa et al. 2002 [31]. The plate was sensitized with 100 µL/well of a venom solution in PBS (10 µg/mL w/v), followed by incubation overnight at 4 °C. Afterward, the wells were washed twice with 200 µL of washer buffer (PBS/Tween 0.05%) and a 100 µL of blocking solution (PBS/BSA 5%) was added, followed by incubation at 37 °C for 2 h. The plate was washed and 100 µL/well of each pre-diluted serum (PBS/BSA 0.1%) were added and then incubated at 37 °C for 1 h. Subsequently, 100 µL/well of conjugated antibodies were added and the plate was incubated at 37 °C for 1 hour. The plate was washed and 50 µL/well of diluted detection antibodies was added and incubated for 3 h. The plate was then washed again, the substrate was added, and the plate was incubated at room temperature for 15 min. The reaction was stopped (H_2_SO_4_ 4 M) and the plate was read at 450 nm.

### Statistical analysis

The results are expressed as mean ± standard deviation. Statistical analyses were realized using one-way analysis of variance (ANOVA) with Tukey’s test using GraphPad Prism version 5.00 (San Diego, CA, USA). Differences in the mean values with *** or ^###^*p* < 0.001, ** or ^##^*p* < 0.01, or * or ^#^*p* < 0.05 were considered statistically significant.

## Data Availability

All data that supports the findings of this study is available in the published article and/or the supporting information of this article.
